# Supplementary material for: Cortical modulation of sensory flow during active touch in the rat whisker system
Source: Nat Commun. 2018 Sep 25;9:3907. doi: 10.1038/s41467-018-06200-6 (PMC6156333; doi:10.1038/s41467-018-06200-6)
Supplement: Supplementary file 1 — Supplementary Information [file 41467_2018_6200_MOESM1_ESM.pdf]

Supplementary Information

Cortical modulation of sensory flow during active touch in the rat whisker system

Chakrabarti & Schwarz

Supplementary Figure 1

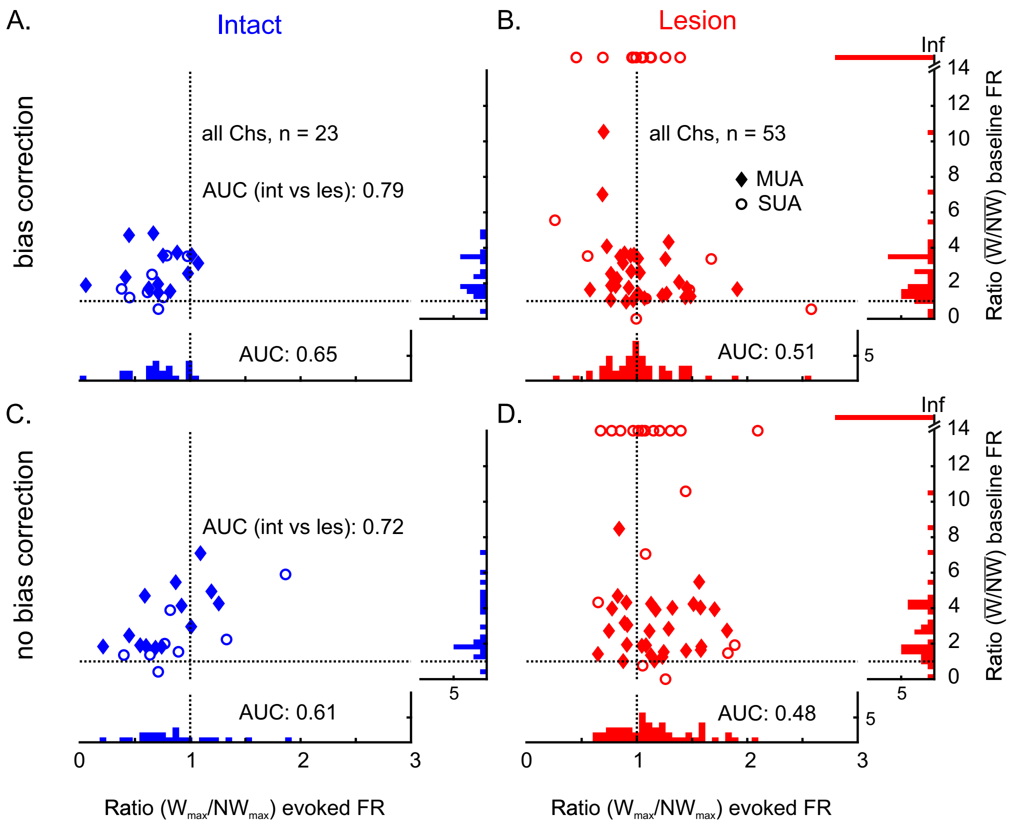

Supplementary Figure 1: The effect of specific trial selection on sensory gating. **A-B.** Scatterplots showing evoked and baseline firing rate ratios of whisking and non-whisking trials, identical to plots in Figure 2-3C obtained using a selection of trials with overlapping distributions of contact induced whisker decelerations. **C-D.** Same analysis performed by taking all trials.

Supplementary Figure 2

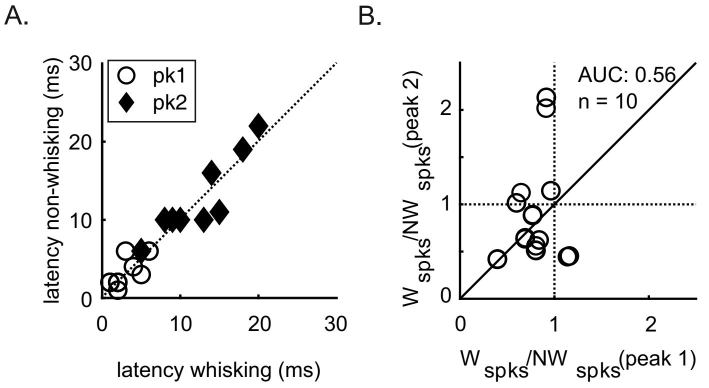

Supplementary Figure 2: Latencies of peak responses in whisking and non-whisking trials. **A.** The latencies in ms of the first and second peaks (see methods) for whisking (abscissa) and non-whisking (ordinate) trials for all 10 neurons that exhibited two peaks. Open circles and filled diamonds indicate first and second peaks, respectively. Diagonal indicates the unity line. **B.** Sensory gating (ratios of whisking and non-whisking spike numbers) for each peak. Values less than 1 (dotted lines) on both axes denote sensory gating. Effect size (AUC) calculated between the ratios of first and second peaks.

Supplementary Figure 3

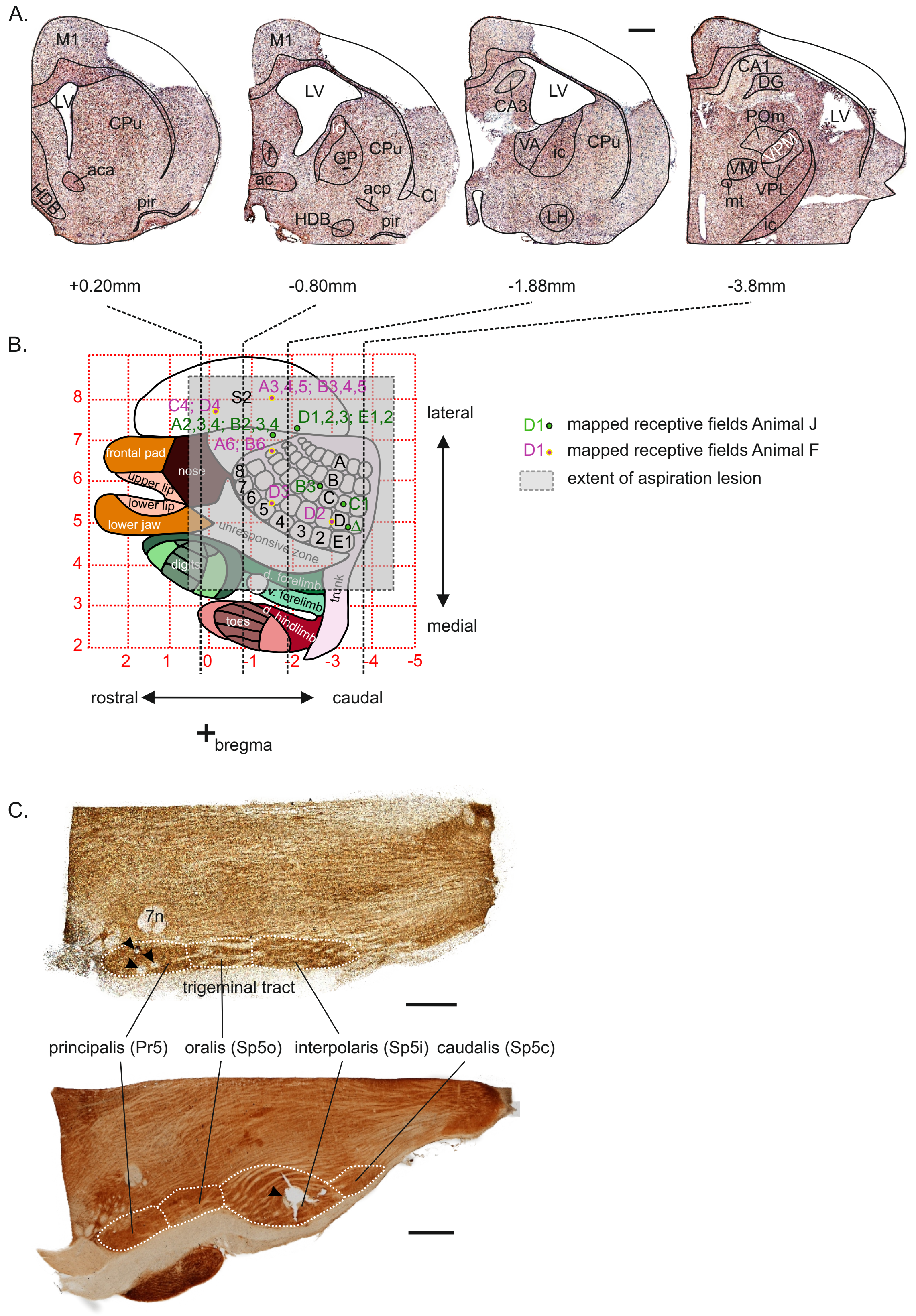

Supplementary Figure 3: Cortical lesions. **A.** An example of a cortical aspiration lesion shown using coronal sections from different rostrocaudal levels. Sections stained using Haematoxylin-Eosin mixture. **B.** Each section shown superimposed on a map of S1 and S2 73 representations with stereotaxic coordinates superimposed. Gray region shows the extent of the aspiration lesion in both animals whereas the magenta and green dots with accompanying notation denote electrode penetrations made in each animal and receptive fields encountered. **C:** Recording locations in brainstem in two representative cases showing electrode penetrations in Pr5 (top) and Sp5i (bottom). The animal on the top panel is the same one shown in A. Scalebars : 1mm.

Supplementary Figure 4

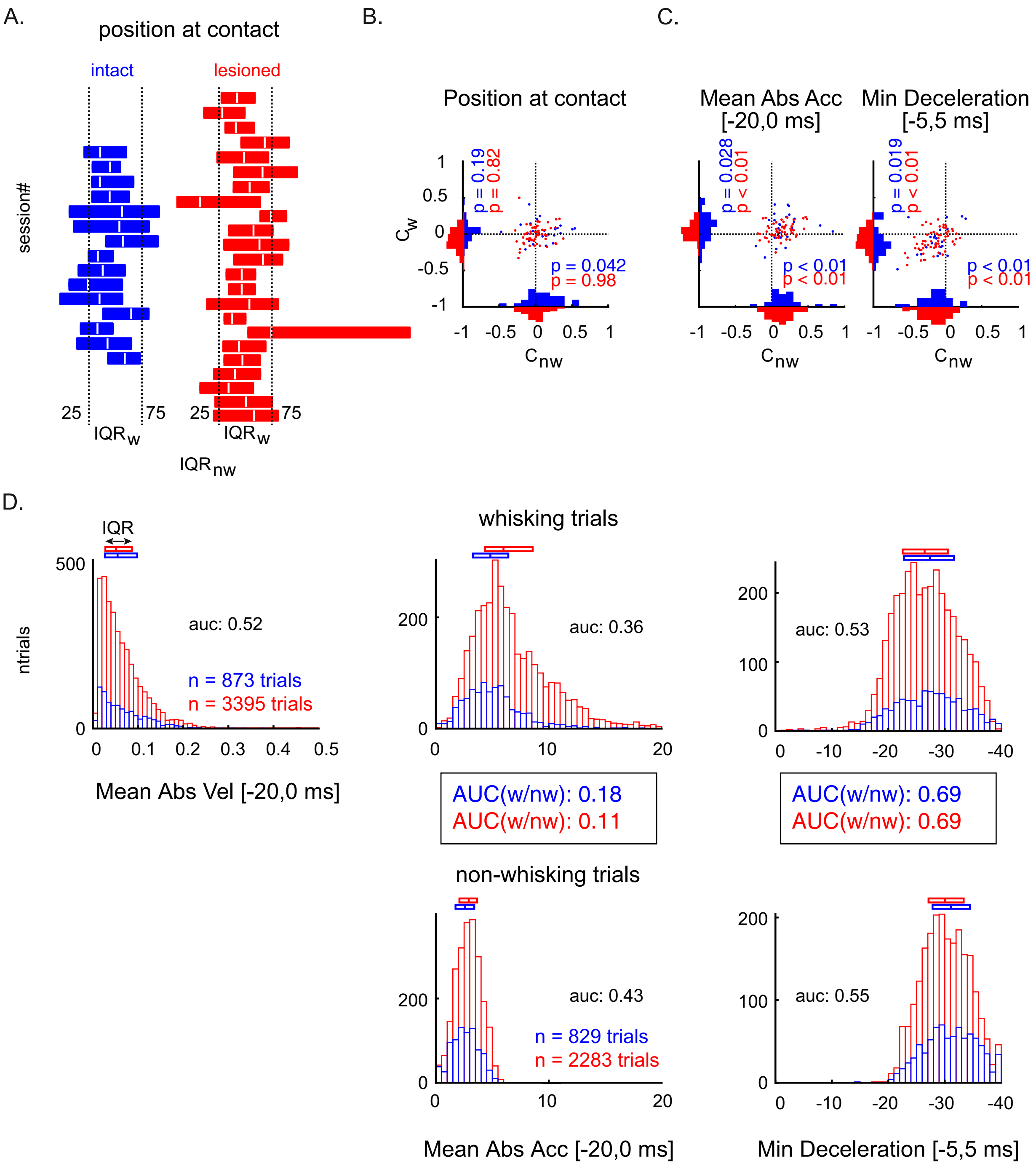

Supplementary Figure 4: Whisking kinematics . **A.** Medians (white vertical lines) and interquartile ranges (colored boxes) of whisker positions (set points) at contact during non-whisking trials for each session, normalized to the 25th and 75th percentiles (vertical dotted lines) of whisker positions from whisking trials in the same session. Blue and red show identical analyses for intact and lesioned animals, respectively. IQR: Interquartile range. **B-C.** The relationship between Pr5 spiking and kinematic parameters such as whisker position at contact (B), mean absolute pre-contact acceleration (C, left panel) and minimum peri-contact deceleration (C, right panel). Scatterplots show correlation coefficients computed between spike counts (0-10ms) and the respective kinematic parameters for each neuron on a trial by trial basis for whisking (ordinate) and non-whisking (abscissa) trials separately. Correlation coefficients centered around zero show little or no encoding of that particular kinematic feature by spike counts in Pr5. Identical analyses for intact (blue) and lesioned (red) animals. p values obtained by testing each distribution against zero (Wilcoxon sign rank test). **D.** Distributions of kinematic parameters such as pre-contact velocities (left), mean absolute pre-contact acceleration (middle) and minimum peri-contact deceleration (right) in intact (blue) and lesioned (red) animals for whisking (top row) and non-whisking (bottom row) trials separately. Pre-contact velocity distributions for non-whisking trials are not shown as they were zero by definition (see methods). Effect sizes (AUC) calculated between lesioned and intact distributions shown on each figure whereas those computed between whisking and non-whisking trials shown in boxes between the rows.

Supplementary Figure 5

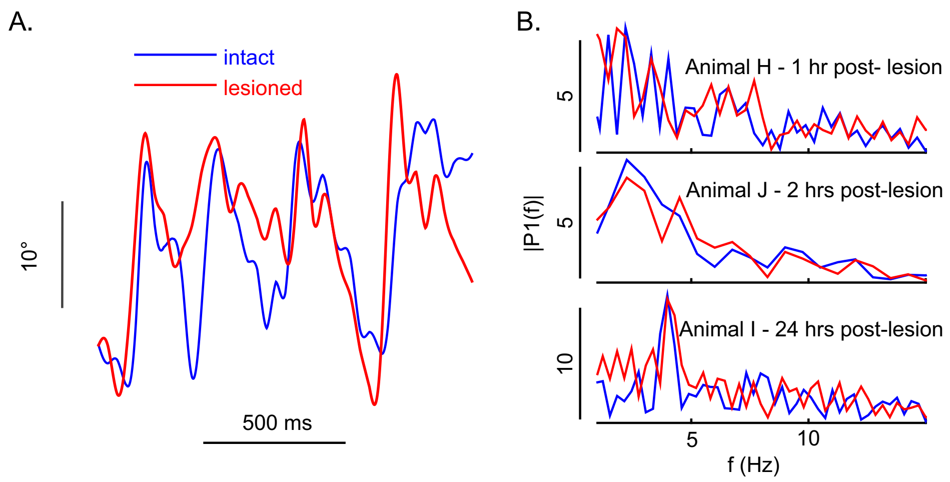

Supplementary Figure 5: A. Whisker traces extracted from video recordings of the freely moving animal (2 hrs after lesion) contralateral to the lesioned (red) and intact (blue) hemispheres. **B.** Power spectra for the three lesioned animals recorded at different time points after the lesion surgeries.

Supplementary Figure 6

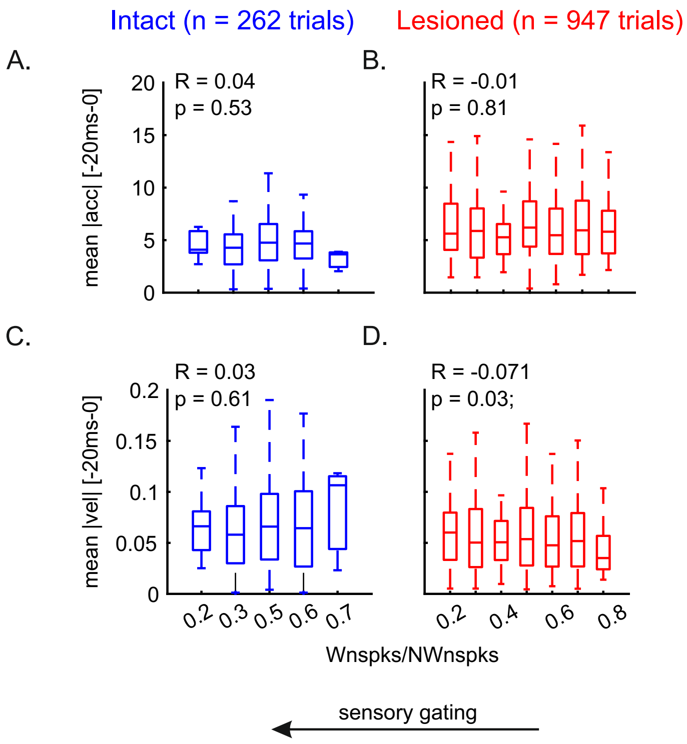

Supplementary Figure 6: Sensory gating and whisking kinematics **A-C.** Trial by trial mean absolute pre-contact accelerations (**A**) and velocities (**C**) against binned evoked firing rate ratios between whisking and non-whisking trials. Only trials showing sensory gating (Wnspks/NWnspks < 1) have been shown. Spearmann's correlation coefficients with accompanying p values indicated in each panel. **B-D.** Identical analysis for lesioned animals. Boxes show interquartile ranges, horizontal tick medians and whiskers the most extreme data points.
